# Abnormal patterns of corticomuscular and intermuscular coherence in childhood dystonia

**DOI:** 10.1016/j.clinph.2020.01.012

**Published:** 2020-04

**Authors:** Verity M. McClelland, Zoran Cvetkovic, Jean-Pierre Lin, Kerry R. Mills, Peter Brown

**Affiliations:** a Department of Basic and Clinical Neuroscience, Institute of Psychiatry, Psychology and Neuroscience, King’s College London, United Kingdom; b Children’s Neurosciences Department, Evelina London Children’s Hospital, Guy’s and St Thomas NHS Foundation Trust, London, United Kingdom; c Department of Informatics, King’s College London, United Kingdom; d Medical Research Council Brain Network Dynamics Unit and Nuffield Department of Clinical Neurosciences, University of Oxford, United Kingdom

**Keywords:** Dystonia, Children, Sensorimotor integration, Corticomuscular coherence, Intermuscular coherence, CMC, corticomuscular coherence, EEG, electroencephalogram, EMG, electromyogram, IMC, intermuscular coherence, FDI, First Dorsal Interosseous, FExt, Forearm Extensors, DBS, Deep Brain Stimulation, BFMDRS-m, Burke-Fahn-Marsden Dystonia Rating Scale motor score, MVC, maximum voluntary contraction, IQR, inter-quartile range, ERD, Event Related Desynchronisation, ERS, Event Related Synchronisation, CV, Coefficient of Variation

## Abstract

**Objective:**

Sensorimotor processing is abnormal in Idiopathic/Genetic dystonias, but poorly studied in Acquired dystonias. Beta-Corticomuscular coherence (CMC) quantifies coupling between oscillatory electroencephalogram (EEG) and electromyogram (EMG) activity and is modulated by sensory stimuli. We test the hypothesis that sensory modulation of CMC and intermuscular coherence (IMC) is abnormal in Idiopathic/Genetic and Acquired dystonias.

**Methods:**

Participants: 11 children with Acquired dystonia, 5 with Idiopathic/Genetic dystonia, 13 controls (12–18 years). CMC and IMC were recorded during a grasp task, with mechanical perturbations provided by an electromechanical tapper. Coherence patterns pre- and post-stimulus were compared across groups.

**Results:**

Beta-CMC increased post-stimulus in Controls and Acquired dystonia (p = 0.001 and p = 0.010, respectively), but not in Idiopathic/Genetic dystonia (p = 0.799). The modulation differed between groups, being larger in both Controls and Acquired dystonia compared with Idiopathic/Genetic dystonia (p = 0.003 and p = 0.022). Beta-IMC increased significantly post-stimulus in Controls (p = 0.004), but not in dystonia. Prominent 4–12 Hz IMC was seen in all dystonia patients and correlated with severity (rho = 0.618).

**Conclusion:**

Idiopathic/Genetic and Acquired dystonia share an abnormal low-frequency IMC. In contrast, sensory modulation of beta-CMC differed between the two groups.

**Significance:**

The findings suggest that sensorimotor processing is abnormal in Acquired as well as Idiopathic/Genetic dystonia, but that the nature of the abnormality differs.

## Highlights

• Beta-corticomuscular coherence and its modulation by peripheral stimulation is abnormal in dystonia.
• Modulation of beta-corticomuscular coherence differs between idiopathic/genetic & acquired dystonia.
• Strong 4–12 Hz intermuscular coherence is common to idiopathic/genetic & acquired dystonia.

## 1 Introduction

Deep Brain Stimulation (DBS) of the Globus pallidus internus is a well-established management for medically refractory dystonias. The success of pallidal DBS in alleviating painful disabling muscle spasms and improving motor control in dystonia has triggered a rapid expansion in the field of neuromodulation and has intensified interest in the underlying physiology of these conditions, including their developmental aspects (Ismail et al., 2017, Lin and Nardocci, 2016).

For Acquired (formerly Secondary) dystonias, the benefits from DBS are more modest than for Idiopathic/Genetic dystonias without degeneration (formerly Primary dystonia and described here as Idiopathic/Genetic). There is also greater inter-individual variation in outcome and predictive markers are lacking (Koy and Timmermann, 2017, McClelland et al., 2018). The differential effect of DBS on Idiopathic/Genetic versus Acquired dystonias likely reflects differences in underlying pathophysiology between these groups and between individuals. Pathophysiological mechanisms in Idiopathic/Genetic dystonias include reduced inhibition at multiple levels of the central nervous system (Berardelli et al., 1998, Hallett, 2011), exaggerated plasticity (Quartarone et al., 2008), abnormal patterns of basal ganglia neuronal activity (Magarinos-Ascone et al., 2008, McClelland et al., 2016, Starr et al., 2005 2005,, Vitek et al., 1999), and abnormally enhanced synchronised oscillatory activity within the cortex-basal ganglia network (Neumann et al., 2015). Furthermore, and linking the above, there is strong neurophysiological (Frasson et al., 2001, Tinazzi et al., 2000) and imaging (Nelson et al., 2009, Tinazzi et al., 2003) evidence that dystonia is a disorder of sensorimotor integration, with distorted processing of afferent inputs leading to excessive and undesired motor outputs.

The pathophysiology of acquired dystonia is far less thoroughly investigated than that of Idiopathic/Genetic dystonias. Importantly, a few reports have suggested differences in physiology between different categories of dystonia (Kojovic et al., 2013, McClelland et al., 2016, Trompetto et al., 2012). Accordingly, the pathophysiological differences and commonalities between acquired and Idiopathic/Genetic dystonias clearly warrant further study, especially in light of their differential therapeutic response to DBS (Neychev et al., 2011).

Corticomuscular coherence (CMC) is a measure of the synchrony between oscillatory electroencephalogram (EEG) and electromyogram (EMG) activity and reflects bidirectional cortex-muscle interaction (Conway et al., 1995, McClelland et al., 2012a, Witham et al., 2010, Xu et al., 2017). Beta-range CMC is observed particularly during static hold of a compliant object (Graziadio et al., 2010, Kilner et al., 2000) and is modulated by sensory stimuli (McClelland et al., 2012a, Stancak et al., 2005), thus providing a measure of sensorimotor integration. Intermuscular coherence (IMC) reflects a common drive to muscle pairs and is elevated in the 3–7 Hz range in myoclonus dystonia and DYT1 dystonia (Foncke et al., 2007, Grosse et al., 2004). However, CMC has been investigated sparsely in these groups and there is a particular lack of information about these processes in acquired dystonias. This study assesses oscillatory activities relevant to sensorimotor integration by comparing patterns of CMC and IMC and their responsiveness in young people with different aetiological types of dystonia versus healthy controls. In particular, we test the hypothesis that sensorimotor processing, measured by sensory modulation of corticomuscular coherence (CMC) and intermuscular coherence (IMC), differs between Idiopathic/Genetic and Acquired dystonias.

## 2 Methods

### 2.1 Ethical approval

Ethical approval was obtained from the London-Fulham National Research Ethics Committee, London, UK (12/LO/0925). Informed written consent was obtained from the participant or, if under 16-years-old, from parents with assent from the child. The studies were conducted in accordance with the declaration of Helsinki.

### 2.2 Subjects and experimental arrangement

The studies were performed on 16 children with dystonia (11 Acquired, 5 Idiopathic/Genetic, nine female) aged 12–18 years, recruited from the Complex Motor Disorders Service at Evelina London Children’s Hospital, and on 13 healthy children (seven female). The diagnosis and classification of dystonia was confirmed by a consultant paediatric neurologist with specialist expertise in movement disorders (JPL). Severity of dystonia was assessed using the motor score of the Burke-Fahn-Marsden Dystonia Rating Scale (BFMDRS-m) by specialised paediatric therapists, blind to the neurophysiological data.

Subjects were seated comfortably at a table and performed a simple motor task with their dominant hand (13 controls and 11 children with dystonia were right hand dominant by self-report). The task was to hold a 15 cm plastic ruler in a key grip between the thumb and index finger, as described previously (McClelland et al., 2012a). The forearm and wrist were supported to minimise contraction of other muscles and fatigue. Task performance was monitored by the experimenters.

Mechanical perturbations to the task were provided from an electromechanical tapper driven by a power amplifier (Ling Dynamic Systems Limited). Characteristics of the mechanical perturbation have been described in detail previously (McClelland et al., 2012a). In brief, the tapper provided pulses of lateral displacement (1 mm at a velocity of 0.2 m/s) at defined times (not forewarned to the subject), giving the subject the sensation that their grip on the ruler may be lost. The perturbation had a rise-time of 5 ms and duration of 20 ms. A stiff plastic ruler (not a flexible “shatterproof” style ruler) was used, thus minimising any resonance effect following tapping. Stimulus amplitude was constant throughout the experiment and between subjects.

A single trial lasted 5 seconds, with the stimulus delivered 1.1 second after the start of the data collection period. The stimuli were delivered at pseudorandom intervals between 5.6 s and 8.4 s (mean 7 s). Stimuli were delivered and corresponding data epochs collected in blocks of 10–25 (according to child’s ability to maintain performance) with intervening rest periods, up to 200 epochs in total. A maximum voluntary contraction (MVC) of the key grip task was recorded for each subject.

### 2.3 EEG and EMG recording

EMG was recorded using adhesive electrodes placed in a belly-tendon montage over dominant First Dorsal Interosseous (FDI) and Forearm Extensors (FExt). Bipolar EEG was recorded from scalp overlying the contralateral hand area of motor cortex, one electrode positioned 5 cm lateral to the vertex along the interaural line and the other 2.5 cm anterior to it (Halliday et al., 1998, Kristeva et al., 2002, McClelland et al., 2012a). Scalp electrodes were applied using conductive paste, and impedance reduced below 5 kOhm.

### 2.4 Data processing and analysis

EMG and EEG signals were amplified and bandpass filtered (0.5–100 Hz for EEG; 5–250 Hz for EMG). Signals were sampled at 1024 Hz using a Cambridge Electronic Design (CED) Analogue to Digital Converter. The digitised signals were stored and analysed using CED software modified by the authors. Raw EEG signals were reviewed off-line and trials containing movement or blink artefacts rejected. Stimulus-triggered averages of EEGs and rectified EMGs were constructed. The stability of muscle contraction was used as a measure of task performance, thus the mean, standard deviation and coefficient of variation of the level of rectified EMG across all epochs were calculated for each muscle in each subject. The mean level of rectified EMG expressed as a percentage of the rectified EMG during the MVC was also calculated.

Coherence spectra were calculated using non-rectified EMG and EEG signals. Although rectification appears to enhance the detection of CMC at low levels of EMG activity, the extent to which it does so varies with the level of contraction (Farina et al., 2004, Farina et al., 2013, Keenan et al., 2005,). Rectification may therefore have inconsistent effects on coherence across different subjects, muscles and levels of fatigue, whereas CMC calculated using non-rectified EMG is less influenced by these factors (Farina et al., 2013, McClelland et al., 2012b, McClelland et al., 2014, Neto et al., 2010, Neto and Christou, 2010, Stegeman et al., 2010).

#### 2.4.1 Coherence

**Fig. 1:**
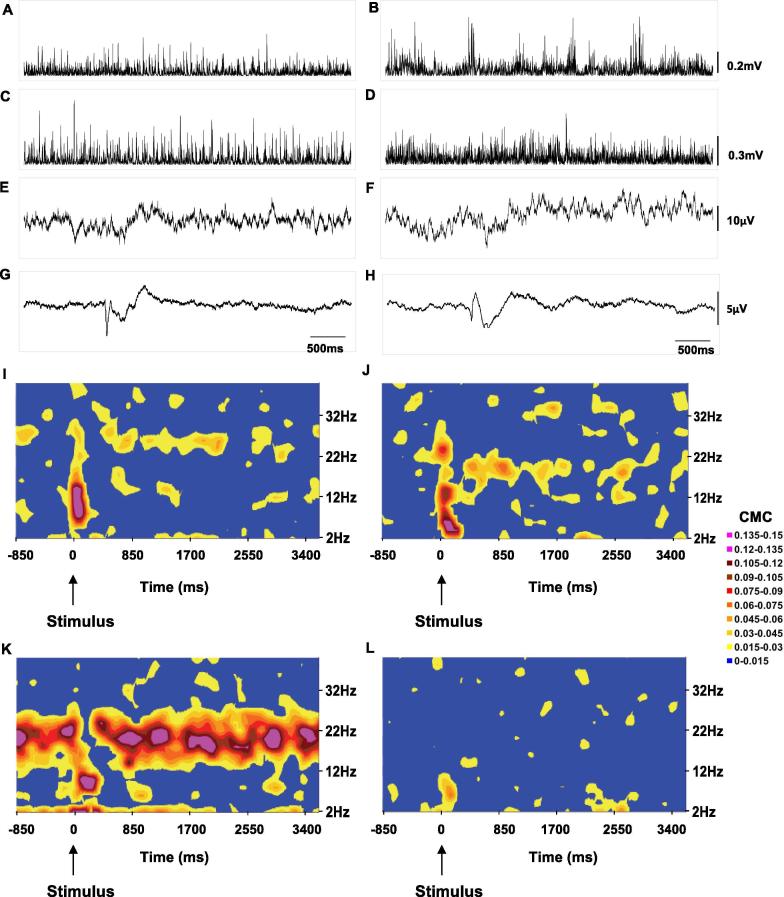
Individual data from control subject (Subject 7) (left column) and child with acquired dystonia (Subject 21) (right column). (A-B): Rectified first dorsal interosseous (FDI) electromyogram (EMG), (C-D): Rectified forearm extensor (FExt) EMG, (E-F): Raw dominant hemisphere electroencephalogram (EEGD), (G-H): Averaged Evoked Potential. Vertical scale bars for left and right columns are equivalent. Horizontal (time) scales for A-H are equivalent. (I-J): Spectrograms from same individuals showing FExt:EEGD coherence (colour scale) at each frequency (y-axis) over time (x-axis) with respect to the stimulus (arrow). Blue represents non-significant coherence values. Corticomuscular coherence (CMC) is estimated in overlapping 500 ms windows plotted for each 50 ms step. Specified time always refers to the mid-point of the 500 ms time windows. Note patient in J shows clear CMC post-stimulus but not at baseline. K-L. Further individual spectrograms from another control (Subject 12) (K) with clear CMC both pre and post-stimulus and a child with idiopathic dystonia (Subject 14) (L).

For each subject coherence spectra were computed between paired dominant hemisphere EEG and contralateral EMG signals (Rosenberg et al., 1989), separately for FDI and FExt (CMC), and between the two muscles (IMC). The autospectral and cross-spectral analysis was performed by averaging over disjoint sections of Hanning-tapered data, using a short-time Fourier transform of length 512 points (500 ms), yielding a frequency resolution of 2 Hz. The number of disjoint sections (L) ranged from 102-200 (equivalent to number of epochs). The 500 ms window was moved across the 5-second data epoch in 50 ms steps to assess change in coherence with time in relation to the stimulus. A time-frequency plot (spectrogram) showing the temporal evolution of coherence was constructed for each EMG/EEG or EMG/EMG pair in each subject. (Note the overlapping moving window introduced a smoothing effect to the plots – see Fig. 1). Coherence timings are referred to throughout using the mid-point of the time-window. Coherence was analysed in both the beta (14–38 Hz) and the theta/alpha (4–12 Hz) range. We extended our beta range to 38 Hz as CMC during modest isometric contraction is regularly seen between 30 and 40 Hz (Chakarov et al., 2009, Hansen and Nielsen, 2004, James et al., 2008, McClelland et al., 2012a) and, in the current study, still showed a similar pattern of modulation as in those subjects whose peak frequency was between 14–30 Hz. It was therefore considered to reflect the same physiological phenomenon. The low frequency (4–12 Hz) range was chosen based on previous reports of enhanced low-frequency IMC in adults with genetic or idiopathic dystonias (Foncke et al., 2007, Grosse et al., 2004) and on exaggerated pallidal oscillations in this range which are coherent with dystonic EMG (Barow et al., 2014, Sharott et al., 2008).

The 95% confidence level for the coherence estimates was calculated according to Eq. (1):

(1) $$95\%\;\text{confidence}\;\text{level}\;1-{\left(0.05\right)}^{1/(L-1)}$$

For each individual the coherence was accepted as significant if it exceeded this value in more than 5% of time/frequency bins and was present at a consistent frequency. This analysis was performed separately for the pre-stimulus period and the post-stimulus period. Data from the immediate peri-stimulus period (up to 450 ms post-stimulus) which contained evoked reflex activity in the EMG and possible contamination in the EEG, was excluded since it was considered likely that some of the coherence seen during this time might be artefactual.

Statistical comparisons of coherence values, both between and within subjects were performed using the Fisher transformed coherency for each subject, denoted as ${\hat{z}}_{i}$ and obtained according to Eq. (2):

(2) $${\hat{z}}_{i}=\tanh^{-1}\left|{\hat{R}}_{i}\right|$$

where $\left|{\hat{R}}_{i}\right|$ is the coherency between the *i-*th pair of values. This transformation stabilises the variance (Rosenberg et al., 1989). Where significant IMC was present across a range of frequencies, the phase of the coherence estimate was calculated to assess for possible volume conduction, reflected by zero-phase coherence.

#### 2.4.2 Pooled and individual analysis

**Fig. 2:**
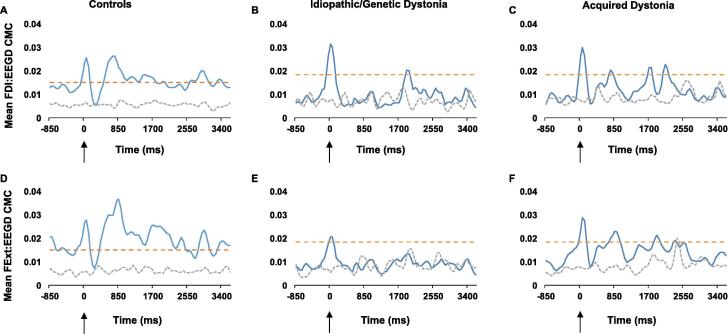
Pooled Frequency Specific beta-corticomuscular coherence (CMC) over time. Mean CMC between First dorsal interosseous EMG and Dominant hemisphere EEG (FDI:EEGD CMC) over time (blue) for (A) Controls (B) Idiopathic/Genetic and (C) Acquired dystonia, using peak-frequency specific data within beta range (14–38 Hz) per individual. Mean FDI:EEGD CMC in a control range (64–88 Hz) is shown for comparison (grey dotted line. Dashed orange line shows 95% confidence level for significant coherence (higher in dystonia groups due to slightly fewer data epochs). CMC is calculated in overlapping 500 ms windows plotted for each 50 ms step. Time scale shows mid-point of each 500 ms data window. Arrow indicates time of tap stimulus. D-F: equivalent figures for CMC between Forearm extensor (FExt) EMG and EEGD (FExt:EEGD CMC).

Figures showing pooled coherence were constructed for each group (Controls, Idiopathic/Genetic Dystonia and Acquired Dystonia) (Amjad et al., 1997, Halliday and Rosenberg, 2000). However, pooling of coherence data has the disadvantage that where the peak frequency of coherence varies considerably between subjects, the individual patterns of significant coherence are blurred or obscured. Analyses were therefore performed based on the peak frequency of beta-coherence per individual (McClelland et al., 2012a). For each subject, the peak frequency of beta-range (14–38 Hz) coherence was identified as the frequency bin with the maximum mean coherence across the whole post-stimulus period. Coherence values for each subject using this peak frequency bin and one bin either side were then pooled for each time point, to establish how the peak beta-coherence changed over time (Fig. 2). The same procedure was followed for a control frequency range (64–88 Hz).

#### 2.4.3 Pre- and post-stimulus comparisons

Assessment of the modulation of coherence during the task was based on the time-course of CMC modulation documented previously (McClelland et al., 2012a), which typically comprises a brief decrease in beta-CMC immediately following the stimulus, followed by an increase in beta-CMC in the early post-stimulus period, peaking between the 450 and 1250 ms post-stimulus windows, followed by a return to baseline levels in the later post-stimulus period. The 5 s epoch length was chosen to ensure enough time for return to baseline, but the key period of interest was the early post-stimulus period. For each individual the mean level of baseline/pre-stimulus coherence at their peak frequency was compared with the mean level of coherence at that frequency for the early post-stimulus (450–1250 ms post-stimulus), and late post-stimulus period (the remainder of the epoch, from 1.25 to 3.6 seconds post stimulus).

#### 2.4.4 Power data and event related synchronisation/desynchronisation

The EEG and EMG power spectra were also analysed separately. The EMG power for each muscle was normalised to the total power across all epochs/trials. The EEG power from dominant sensorimotor cortex was normalised to the total power across all epochs for that individual and analysed in defined frequency bands (Theta 4–8 Hz, Alpha 8–12 Hz, Beta 14–38 Hz), in order to compare absolute levels of EEG power between groups. To assess stimulus-related changes in EEG power over time (Event Related Synchronisation and Desynchronisation), the power for each frequency bin was normalised to the total power in that time window, and then expressed as the percentage change from the mean power in the baseline/pre-stimulus period (Pfurtscheller, 2001). This was analysed for the same frequency bands as above, and across the same time windows. The ratio of beta power to alpha and theta power was also calculated for each time window.

### 2.5 Statistical analyses

Statistical analyses were performed in SPSS Version 25. Comparisons of coherence between groups and between pre- and post-stimulus time periods were made using the individually-specific peak frequency data, using Fisher transformed coherency. The data were assessed for normality using the Shapiro-Wilk test and non-parametric analyses used where applicable.

Our key hypotheses were that levels of beta-CMC and beta-IMC and their pattern of modulation would differ across groups. Levels of beta-CMC and beta-IMC were compared across groups in the baseline and the early post-stimulus periods. Modulation of beta-CMC and beta-IMC in each group was assessed by comparing coherence at baseline with that in the specified early post-stimulus period for each individual. The magnitude of this coherence increase was also compared across groups. The level of 4–12 Hz IMC was compared across groups for the whole epoch. Since data were non-normally distributed, cross-group comparisons were made using the Kruskal Wallis test (two degrees of freedom). Paired pre- versus post-stimulus comparisons were made within each group using the Wilcoxon Signed Rank test (two-tailed). For these a priori hypotheses, the Benjamini-Hochberg procedure was applied with a false discovery rate of 5% to account for multiple comparisons and the cut-off for significance adjusted accordingly.

Where Kruskal Wallis test was significant, post-hoc comparisons were made using the Mann Whitney U test (two-tailed). Secondary, exploratory hypotheses were generated during the analysis relating to changes in spectral power following the stimulus. A significance level of p < 0.05 was accepted for these secondary analyses and for the post-hoc Mann-Whitney analyses.

## 3 Results

Age did not differ significantly between groups (Controls: mean 15.3 years, Dystonia: mean 15 years). Clinical information is given in Table 1. All subjects were able to perform the task, although children with dystonia required more frequent rest periods and guidance. A clear evoked potential was recorded in the contralateral sensorimotor cortex EEG in each subject, (Fig. 1), with no significant difference in amplitude, latency and duration (approximately 200 ms) between groups (see Supplementary Table S1).

**Table 1:** Clinical details.

						Clinical scales		
Case no.	Group	Age at study	Diagnosis	Phenotype	Location of MRI abnormalities	GMFCS	MACS	BFMDRS-m
14	Idiopathic/Genetic	12	Idiopathic	Generalised partially dopa-responsive dystonia	Normal	3	3	8
15	Idiopathic/Genetic	13	Genetic - DYT1 mutation	Generalised dystonia	Normal	1	2	7
16	Idiopathic/Genetic	18	Genetic -KMT2B mutation	Generalised dystonic choreoathetosis with possible myoclonic elements. Whispering dysphonia	BG	2	3	17
17	Idiopathic/Genetic	12	Idiopathic – family history of dopa-responsive dystonia	Generalised dystonia-dyskinesia	Normal	1	3	12
18	Idiopathic/Genetic	17	Genetic – TITF1 mutation	Generalised dystonia with myoclonus	Normal	1	2	9
19	Acquired	12	Cerebral palsy secondary to Perinatal HIE	Generalised dystonia-dyskinesia	BG, WM, Cortex	4	4	27
20	Acquired	15	Cerebral palsy secondary to Perinatal HIE	Generalised dystonia-dyskinesia	WM	1	2	10
21	Acquired	15	Cerebral palsy secondary to perinatal HIE	Generalised dystonia-dyskinesia	Normal	2	2	8
22	Acquired	18	Unknown. Mild white matter changes on MRI	Generalised dystonia onset age 13.	WM	1	1	7
23	Acquired	15	Presumed perinatal injury	Asymmetric dystonia Right > Left	BG, WM, Cortex	2	3	N/A
24	Acquired	17	Perinatal arrested hydrocephalus	Asymmetric dystonia Right > Left	BG, WM	2	2	7
25	Acquired	17	Right Middle Cerebral Artery infarct	Asymmetric dystonia Left > Right	BG, WM, Cortex	1	2	N/A
26	Acquired	14	Glutaric aciduria with symmetrical gliosis of putamina bilaterally.	Dystonia + choreoathetosis	BG	2	3	N/A
27	Acquired	18	Unknown	Early onset dystonia from 11 months. Severe expressive language difficulties	BG	5	4	N/A
28	Acquired	13	Cerebral palsy secondary to perinatal HIE	Dystonia + athetosis	BG	2	2	14
29	Acquired	14	Cerebral palsy secondary to perinatal HIE	Dystonia + athetosis	BG, WM	2	2	12

MRI – Magnetic Resonance Imaging.

BG – Basal Ganglia.

WM – White Matter.

GMFCS – Gross Motor Function Classification System score.

MACS – Manual Ability Classification System score.

BFMDRS – Burke Fahn Marsden Classification System – motor score.

HIE – Hypoxic Ischaemic Encephalopathy.

N/A – not available.

### 3.1 Beta-corticomuscular coherence (CMC)

Numbers of children showing significant beta-range CMC at baseline and post-stimulus are given in Table 2 (group data) and Supplementary Table S2 (individual data). Example spectrograms are shown in Fig. 1. Pooled peak frequency data are shown in Fig. 2.

**Table 2:** Summary of number of individuals showing beta range corticomuscular and intermuscular coherence.

Group	FDI:EEGD Corticomuscular Coherence			FExt:EEGD Corticomuscular Coherence			FDI:FExt Intermuscular Coherence		
	Baseline	Post-stimulus	Peak Frequency (Hz)	Baseline	Post-stimulus	Peak Frequency (Hz)	Baseline	Post-stimulus	Peak Frequency (Hz)
Control	7/13 (54%)	12/13 (92%)	Median 22 Range 14–36 IQR 16	9/13 (69%)	12/13 (92%)	Median 24 Range 18–32 IQR 10	6/13 (46%)	13/13 (100%)	Median 22 Range 14–36 IQR 6
Idiopathic/Genetic Dystonia	1/5 (20%)	3/5 (60%)	Median 24 Range 22–30 IQR 7	1/5 (20%)	3/5 (60%)	Median 26 Range 24–30 IQR 5	4/5 (80%)	5/5 (100%)	Median 22 Range 18–28 IQR 8
Acquired Dystonia	3/11 (27%)	8/11 (73%)	Median 22 Range 16–30 IQR 8	5/11 (45%)	9/11 (82%)	Median 22 Range 20–36 IQR 16	8/11 (73%)	11/11 (100%)	Median 26 Range 20–30 IQR 4

FDI – First dorsal interosseous.

FExt – Forearm extensors.

EEGD – Dominant hemisphere EEG.

IQR – Interquartile range.

Compared with controls, fewer children with dystonia showed beta-CMC in the baseline period. In the post-stimulus period, all subjects showed CMC in one or both muscle:EEG combinations. When present, beta-CMC was generally weaker in dystonia than in Controls, particularly in Idiopathic/Genetic dystonia (Fig. 1L, Fig. 2B&E). The Acquired dystonia group showed mean levels of beta-CMC intermediate between Controls and the Idiopathic/Genetic group (Fig. 2C&F).

The magnitude of beta-CMC did not differ significantly between the three groups at baseline (Kruskal-Wallis H = 0.007, p = 0.997), but did differ significantly in the early post-stimulus period (Kruskal-Wallis H = 9.633, p = 0.008) with the Idiopathic/Genetic group showing significantly reduced levels of CMC compared with Controls (Mann-Whitney U = 50.0, p = 0.002). The Idiopathic/Genetic group also showed significantly lower levels of CMC compared with the Acquired group (Mann-Whitney U = 52.0, p = 0.039).

### 3.2 Beta-intermuscular coherence (IMC)

**Fig. 3:**
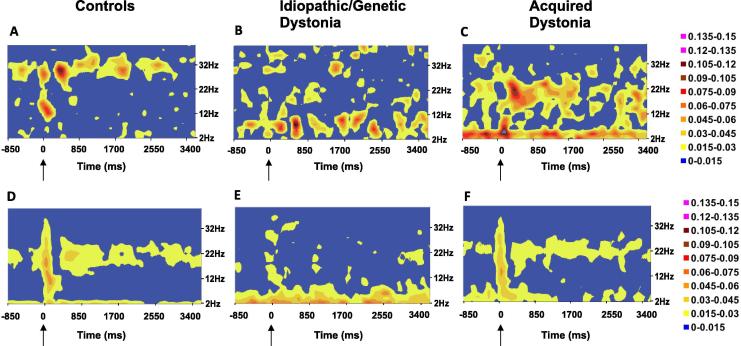
Individual and Pooled Spectrograms for Intermuscular coherence (IMC). Top row: Example spectrograms from individual subjects from (A) Control (Subject 13), (B) Idiopathic/Genetic (Subject 14) and (C) Acquired (Subject 21) dystonia groups, showing IMC (colour scale) at each frequency (y-axis) over time (x-axis) with respect to the stimulus (arrow). Blue represents non-significant coherence values. IMC is estimated in overlapping 500 ms windows plotted for each 50 ms step. Specified time always refers to the mid-point of the 500 ms time windows. Bottom row: Pooled spectrograms for (D) controls, (E) Idiopathic/Genetic and (F) Acquired dystonia. Note the prominent band of 4–12 Hz IMC seen in both dystonia groups, but absent in controls.

Numbers of children showing significant beta-range IMC are given in Table 2 (group data) and Supplementary Table S2 (individual data). IMC spectrograms are shown in Fig. 3. The peak frequency of beta-IMC was not necessarily the same as the peak frequency of CMC for a given individual (Supplementary Table S2). In contrast to beta-CMC, there was less variability between individuals in the peak frequency of beta-IMC, which can therefore be visualised clearly in the pooled spectrograms. Significant beta-IMC was observed in all subjects in the post-stimulus period. Beta-IMC was seen less consistently in Idiopathic/Genetic compared with Controls or Acquired dystonia (Fig. 3), but at group level, the magnitude of beta-IMC was not significantly different, either at baseline (Kruskal-Wallis H = 3.103, p = 0.212) or during the early post-stimulus period (Kruskal-Wallis H = 1.215, p = 0.545).

### 3.3 Modulation of Beta-CMC

**Fig. 4:**
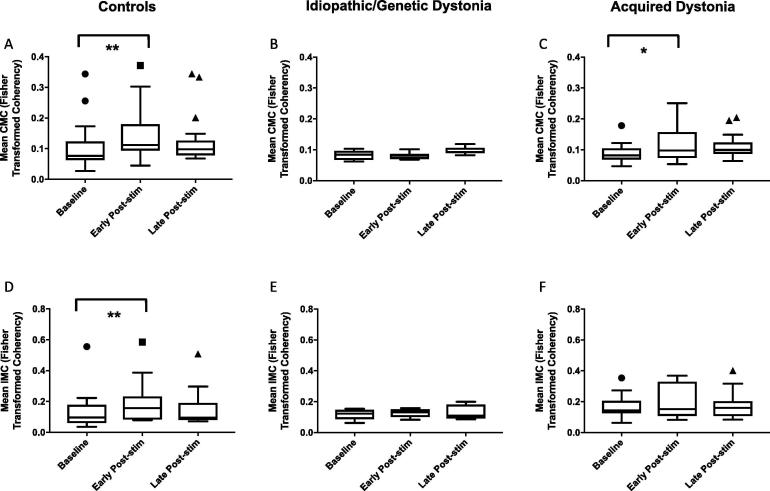
Change in beta-coherence from pre-to post-stimulus. Tukey Box-plots show median and interquartile range for each group of the Fisher transformed peak-frequency-specific coherence for each individual in the pre-stimulus (1100 to −100 ms with respect to the stimulus), early (0.2–1 second post stimulus) and late (1–3.5 seconds post-stimulus) post-stimulus periods. Whiskers show 75th centile plus 1.5 Inter-quartile range (IQR) and 25th centile minus 1.5 IQR. Outliers beyond these points are shown as individual values. Top row: Corticomuscular coherence (CMC) (First dorsal interosseous (FDI) EMG:dominant hemisphere EEG and Forearm extensor (FExt) EMG:dominant hemisphere EEG averaged for each group); Bottom row: Intermuscular coherence (IMC). *p < 0.05 **p < 0.01.

In Controls, the magnitude of beta-CMC increased significantly from baseline to early post-stimulus (Wilcoxon signed ranks Z = −3.365, p = 0.001), peaking between 450–1250 ms and returning to baseline in the later post-stimulus period (Fig. 4A), consistent with adult findings (McClelland et al., 2012a). In contrast to Controls, the Idiopathic/Genetic group did not show a significant increase in beta-CMC post-stimulus (Wilcoxon signed ranks Z = −0.255, p = 0.799) (Fig. 4B). The Acquired group, however, showed a pattern similar to Controls with a significant increase in beta-CMC post-stimulus (Wilcoxon signed ranks Z = 2.581 p = 0.010) (Fig. 4C). The increase in magnitude of beta-CMC from baseline to early post-stimulus was significantly different between groups (Kruskal Wallis H = 8.164, p = 0.017), with the increase being significantly larger in Controls than Idiopathic/Genetic dystonia (Mann-Whitney U = 49.0, p = 0.003) and significantly larger in Acquired than Idiopathic/Genetic dystonia (Mann-Whitney U = 54.0, p = 0.022). There was no significant difference in this measure between Controls and Acquired. Comparisons of CMC patterns across different clinical phenotypes and different aetiologies within the Acquired group are included in the Supplementary Information.

### 3.4 Modulation of Beta-IMC

As with CMC, the magnitude of beta-IMC in Controls increased significantly from baseline to early post-stimulus (Wilcoxon Signed Ranks Z = −2.900, p = 0.004) (Fig. 4D). In contrast, beta-IMC did not increase significantly from baseline to early post-stimulus in either the Idiopathic/Genetic (Wilcoxon Signed Ranks Z = −0.674, p = 0.500) or Acquired groups (Wilcoxon Signed Ranks Z = −1.245, p = 0.213) (Fig. 4E–F). However, overall the magnitude of the increase between baseline and early post-stimulus did not differ significantly between groups (Kruskal-Wallis test: H = 2.102, p = 0.350).

### 3.5 Low-frequency intermuscular coherence

**Fig. 5:**
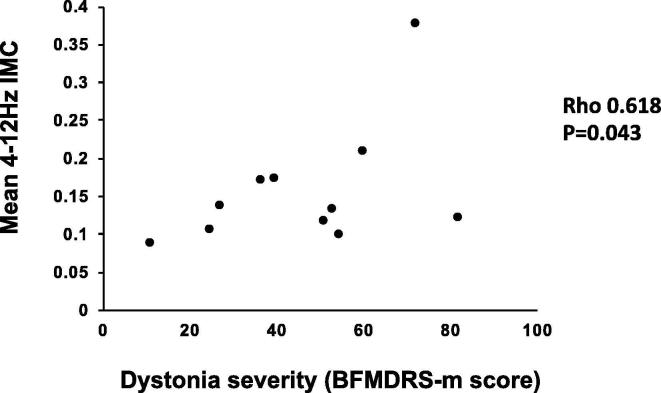
Relationship of intermuscular coherence (IMC) to SeverityDystonia severity, measured as Burke-Fahn-Marsden Dystonia Rating Scale motor score, plotted against mean IMC across 4–12 Hz band. Results of Spearman Correlation are shown.

All children with dystonia showed significant 4–12 Hz IMC (Fig. 3). This was not seen consistently in Controls. The difference between groups in the median amplitude of 4–12 Hz IMC across the entire epoch was statistically significant (Kruskal Wallis H = 13.283, p = 0.001). Post-hoc tests confirmed the amplitude of 4–12 Hz IMC was significantly higher in both dystonia groups compared with Controls (Controls versus Idiopathic/Genetic: Mann-Whitney U = 9.0, p = 0.019; Controls versus Acquired dystonia: Mann-Whitney U = 12.0, p = 0.000217). There was no significant difference in the level of 4–12 Hz IMC between the two dystonia groups. Phase spectra demonstrated a change in phase with frequency, consistent with non-zero phase difference in which FExt led FDI (see Supplementary Fig. S1). Dystonia severity, assessed using BFMDRS-m, was available in 11/16 children and ranged from 11 to 82 (median 51). Fig. 5 demonstrates a correlation between severity and low frequency IMC (Spearman Rho 0.618, p = 0.043).

### 3.6 EEG power and event related synchronisation/desynchronisation

Mean normalised EEG power across the whole epoch did not differ significantly between groups in the theta, alpha or beta range. Stimulus–related changes in EEG power are shown for each group in Supplementary Fig. S2. Controls showed a well-defined decrease in beta power (Beta-Event-related desynchronization, ERD) with negative peak consistently between 150 and 350 ms post-stimulus (mid-point of time window) followed by an increase (Beta-Event-related synchronisation, ERS), with peak between 500 and 950 ms post-stimulus, and then a return to baseline by approximately 1 second post-stimulus (Fig. S2A). The Beta-ERD was accompanied by an ERS in the theta-alpha range, with return to baseline by approximately 1 second post-stimulus (Fig. S2D).

The beta-ERD in Idiopathic/Genetic dystonia was similar to Controls, whereas the magnitude of beta-ERD in Acquired dystonia was smaller than in Controls (Mann-Whitney U = 36.0, p = 0.041). However, the beta-ERD magnitude did not differ significantly between Idiopathic/Genetic and Acquired dystonia. The clear beta-ERS identified in Controls showed a more variable time course in both dystonia groups. The pattern of beta-ERD in relation to beta-CMC modulation for each individual is shown in Supplementary Table S3.

The ratio of beta power to theta-alpha power over time was plotted for each individual (Fig. S2G-I). All groups showed a decrease in beta/theta-alpha ratio in response to the stimulus but, while the Control and Idiopathic/Genetic groups returned quickly to baseline, the ratio in the Acquired group showed more prolonged fluctuations extending into the late post-stimulus period. This was quantified by calculating the Coefficient of Variation (CV) of the beta/theta-alpha ratio over time in each subject using the same time periods defined for coherence analysis: baseline, early and late post-stimulus. The CV in the late post-stimulus period was significantly different between groups (Median CV Controls 0.073, Primary 0.070, Acquired 0.113, Kruskal Wallis H = 8.849, p = 0.012), with post-hoc test showing a significantly higher CV for Acquired dystonia compared with Controls (Mann-Whitney U = 18.0, p = 0.001). The difference in CV between Genetic/Idiopathic and Acquired was not significant (Mann-Whitney U = 16.0, p = 0.221). Importantly, the CV did not differ significantly between groups for the baseline period (Median CV Controls 0.047, Primary 0.085, Acquired 0.070, Kruskal Wallis H = 2.356, p = 0.308).

### 3.7 Were coherence or ERD/ERS findings confounded by more variability in task performance or tremor in dystonia?

The task involved isometric contraction. At group level, individuals with dystonia generally used a higher percentage of MVC to perform the task compared with Controls (Supplementary Table S4a and S4b). Stability in the level of muscle contraction was used as a measure of task performance. The coefficient of variation (CV) of the level of rectified EMG was not significantly different between groups for either muscle (Median CV for FDI: Controls 1.301, Idiopathic/Genetic 1.237, Acquired 1.295, Kruskal Wallis H = 1.843, p = 0.398. Median CV for FExt: Controls 0.954, Idiopathic/Genetic 1.041, Acquired 1.130, Kruskal Wallis H = 2.780, p = 0.249).

There was no significant correlation between the percentage of MVC or the CV of the level of rectified EMG and the mean amplitude of beta-CMC or beta-IMC seen in each individual (see Supplementary Table S5a). Furthermore, there was no correlation between the level of low frequency IMC and the CV (Supplementary Table S5b). In view of the potential confounding effect of differences in both the level of MVC and the level of low frequency IMC between groups, partial correlation analysis was performed which confirmed there was no correlation between the level of MVC and the level of low frequency IMC (Supplementary Table S5b). It is therefore unlikely that the differences in CMC and IMC observed between groups could be explained by differences in muscle activation or task performance. Furthermore, there was no clear rhythmic EMG bursting pattern consistent with tremor activity (see Fig. 1).

Finally, there was no correlation between the timing of the ERD and ERS peaks and the return to baseline of the EEG activity following the cortical evoked potential or the level of on-going movement as reflected by the coefficient of variation of the EMG.

## 4 Discussion

Abnormal motor unit synchronisation between antagonist muscles is a recognised feature of dystonia (Farmer et al., 1998), implying an abnormal descending drive, which may not necessarily be cortical in origin. However, few studies in dystonia have investigated coherence between cortex and muscle directly. Furthermore, most studies have focused on primary (Idiopathic or Genetic) dystonias. The current study provides novel findings by investigating both CMC and IMC in the beta and theta-alpha range in both primary (Idiopathic/Genetic) and Acquired dystonia. The paradigm used facilitates detection of CMC and provides a measure of sensorimotor integration, by assessing CMC modulation in response to a relevant sensory stimulus. The key and novel findings are:

• In typically developing children both beta-CMC and beta-IMC increase following peripheral stimulation then return to baseline, consistent with adult studies (McClelland et al., 2012a) and confirming reproducibility of findings using this paradigm.
• Clear differences in CMC and IMC are seen between controls and children with dystonia: both Acquired and Idiopathic/Genetic dystonia groups show a strong low-frequency (4–12 Hz) IMC which is absent or minimal in controls and correlates with dystonia severity.
  Furthermore, children with Idiopathic/Genetic dystonia show an abnormal pattern of CMC modulation, with significantly lower levels of beta-CMC compared with controls.

• Event related spectral changes are also abnormal in dystonia compared with controls, particularly the amplitude of the beta-ERD which was reduced in Acquired dystonia.
• Further distinctions were identified between Idiopathic/Genetic and Acquired dystonia, with the pattern of beta-CMC modulation differing between the two groups.

### 4.1 The low frequency descending drive in dystonia

A powerful 4–7 Hz muscular drive appears to be a signature of dystonic muscle activity in several patient groups, having been demonstrated in cervical dystonia (Tijssen et al., 2000, Tijssen et al., 2002), DYT1 dystonia (Grosse et al., 2004) and DYT11 myoclonus dystonia (Foncke et al., 2007) but not previously in acquired dystonia (Grosse et al., 2004).

The current study supports and extends these findings. We demonstrate a strong 4–12 Hz IMC in both Idiopathic/Genetic and Acquired dystonia, but not in controls, suggesting this phenomenon is common to dystonia of many different aetiologies. The novel finding of a significant low frequency IMC in Acquired dystonia seen in our study likely reflects the larger sample size and choice of paradigm. We also found a significant correlation of 0.618 between low frequency IMC and dystonia severity (BFMDRS-m). Thus low frequency IMC could predict almost 40% of the variance in BFMDRS-m, highlighting the clinical relevance of this finding. A recent study has also detected a positive correlation between low frequency IMC and dystonia severity in 12 adults, predominantly with cervical dystonia (Doldersum et al., 2019), concordant with our findings. It is also notable that enhanced low frequency pallidal oscillatory activity is coherent with dystonic EMG (Barow et al., 2014, Sharott et al., 2008).

### 4.2 The beta band descending drive in dystonia

The current study demonstrates novel findings in the patterns of beta-range CMC and IMC in dystonia. Foncke et al. (2007) previously reported significant 15–30 Hz coherence between EEG and wrist extensor EMG in 5/13 controls but not in Myoclonus Dystonia. Beta-CMC has not previously been investigated in other types of dystonia. We detected beta-CMC in some children with Idiopathic/Genetic dystonia, but with low magnitude and in brief transient bursts such that overall levels did not change significantly over time, contrasting markedly with controls. The use of a proprioceptive stimulus in the current study enhances levels of CMC (McClelland et al., 2012a). Therefore the observation that children with genetic/idiopathic dystonia did not show strong CMC even following the stimulus is striking. Although this group was small, the inter-individual variability was low (Fig. 4) and their low CMC levels concur with previous literature (Foncke et al., 2007). In contrast, the Acquired dystonia group generally showed beta-CMC patterns similar to controls.

In distinction from the beta-CMC findings, beta-*IMC* was present to a similar degree in controls and dystonia in the post-stimulus period. Whilst beta-IMC appeared stronger in the Acquired than the Idiopathic/Genetic group in the pooled data figures, there was no statistically significant difference in level of beta-IMC or the magnitude of its post-stimulus increase across the three groups.

Cortical oscillations in the beta range are likely to reflect multiple activities, not all relating to descending drive to muscle (Pfurtscheller et al., 1997, Cordivari et al., 2002). This low signal-to-noise ratio results in inevitably low CMC measurements (Xu et al., 2017). IMC is therefore often used as a surrogate of CMC, with cortical drive to muscle being inferred from the pattern of IMC. Our findings show some similarities between IMC and CMC patterns, indicating a degree of overlap, but the differences we observe suggest that EMG:EMG coherence cannot be assumed to be a pure surrogate of CMC. Rather the two methods provide complementary information. This is exemplified by differences in the pattern of abnormality seen between the two. It is interesting that beta-IMC (which may be mediated at least in part by sub-cortical processes) is similar between Idiopathic/Genetic and Acquired dystonia, whereas beta-CMC (which may, to a greater extent, reflect cortical processing) differs between the two groups and is more “abnormal” in Idiopathic/Genetic dystonia. Furthermore, the strong low frequency coherence seen in both dystonia groups was seen only in the IMC and not CMC analysis.

### 4.3 Changes in sensorimotor integration as revealed by spectral reactivity

A striking new observation in this study was the ability (or not) to modulate beta-CMC in response to a peripheral stimulus. Cordivari et al. (2002) previously studied EMG:EMG coherence and its response to electrical digital nerve stimulation in adults with writer’s cramp during a tonic wrist flexion, but found no difference between patients and controls. In the current study, controls showed a strong post-stimulus increase in beta-CMC and beta-IMC, followed by return to baseline, whereas beta-CMC in the Idiopathic/Genetic dystonia group remained unchanged. This failure of modulation is consistent with an abnormality of sensorimotor integration. Transcranial magnetic stimulation and somatosensory evoked potential studies suggest that sensorimotor integration is disrupted in idiopathic and genetic dystonias (Avanzino et al., 2015, Frasson et al., 2001, Tinazzi et al., 2000, Tinazzi et al., 2003). We provide further evidence to support this concept in a paradigm linking sensorimotor integration with cortical oscillatory activity.

The lack of CMC modulation in our Idiopathic/Genetic group was observed despite these individuals showing normal evoked potentials and normal initial event-related spectral EEG changes. These findings suggest that afferent information was received at the sensorimotor cortex and underwent initial sensory processing. However, these patients appeared not to utilize this information in producing a CMC response. In contrast to Idiopathic/Genetic dystonia, modulation of beta-CMC was preserved in many patients with Acquired dystonia (Supplementary Table S3). At group level, the difference in magnitude of beta-CMC modulation was statistically significant, indicating a divergence in sensorimotor processing between Idiopathic/Genetic and Acquired dystonia.

Few other studies have investigated cortical oscillatory activity in acquired dystonia. Kukke et al. found that spectral changes in sensorimotor cortex EEG during a motor task were reduced in the ipsilesional hemisphere of patients with hemidystonia following childhood stroke (Kukke et al., 2015). Our own findings complement these, since our Acquired dystonia group showed abnormal ERD/ERS patterns. Aberrant ERD/ERS patterns have been reported in children with spastic cerebral palsy, (Riquelme et al., 2014, Kurz et al., 2015a, Kurz et al., 2015b), but neurophysiological studies in dystonic cerebral palsy remain sparse.

Overall, these observations demonstrate a difference in the pattern of cortical oscillatory activities and their relationship with muscle activity, not only between controls and children with dystonia but also between patients with Acquired and Idiopathic/Genetic dystonia. They also illustrate differences between patients *within* the Acquired dystonia group (see also Supplementary File). A possible interpretation is that whilst both groups show an abnormality of sensorimotor integration/processing, the point in this process (or node in the network) at which the abnormality arises may be different between individuals, depending on the aetiology and/or timing of the brain insult. We raised this notion previously after demonstrating that even the initial arrival of afferent information (reflected by median nerve somatosensory evoked potential N20 component), is abnormal in approximately 50% of patients with Acquired dystonia (McClelland et al., 2018). We now demonstrate that in a subset of Acquired dystonia patients, the afferent information is received but not processed normally (as represented by initial beta-ERD). In contrast, the children with Idiopathic/Genetic dystonia receive and initially process the information but this is not integrated into the motor drive*.*

### 4.4 Acquired dystonia considerations

Acquired dystonias are much more common than genetic or idiopathic dystonias in childhood but there is little published research concerning their pathophysiology. Preliminary analyses comparing sub-groups of the Acquired dystonia group in the current study (see Supplementary Information) suggest possible differences in patterns of beta-CMC and its modulation across different aetiologies and/or phenotypes of dystonia. However, cautious interpretation is needed in view of the smaller numbers in these sub-groups and further, larger studies in this field are warranted.

It should be noted that even within single gene disorders or single aetiologies of acquired dystonia there is wide heterogeneity in clinical presentation, severity and/or response to therapy. For example, Vidailhet et al. (2009) describe a cohort of patients undergoing pallidal Deep Brain Stimulation for dystonia-choreoathetosis cerebral palsy due to neonatal hypoxic ischaemic encephalopathy and report improvements ranging from −7.4% to 55% change in the BFMDRS-m score. It is clear however, that some neurophysiological parameters may cross the boundaries of clinical heterogeneity. In the current study, enhanced low frequency intermuscular coherence was present in the majority of dystonia patients, despite the heterogeneity, showing that this pathological neurophysiological feature is common across different aetiologies of dystonia. Delineating and understanding the commonalities and differences between different types of dystonia, encompassing the clinical and aetiological heterogeneity, is key to understanding both the pathophysiology of the disorder and how therapies such as neuromodulation can be modified and improved on an individualised basis to produce better patient outcomes.

### 4.5 Possible study limitations

• Patient numbers were relatively small, albeit those of Acquired dystonia were larger than previous studies. Therefore only large effects may have been detected.
• The possibility that 4–12 Hz IMC reflects cross-talk is difficult to exclude, but was considered unlikely since this would be expected in all subjects rather than just in patients and the phase of the coherence was non-zero. It is also unlikely to reflect tremor, since rhythmic bursting EMG activity was not seen.
• A single bipolar EEG derivation was used, to minimise the duration of the experiment. Although the location chosen was the one in which maximal CMC is usually detected (Halliday et al., 1998, Kristeva et al., 2002, Graziadio et al., 2010), it is still possible that we missed a maximum CMC elsewhere due to possible cortical re-organisation in patients with acquired dystonia. However, this is unlikely since several patients with perinatally acquired brain lesions still demonstrated strong CMC.
• The dominant hand was used, including some individuals with asymmetric dystonia, because for some patients, the task could not be performed with the more severely affected hand. This may have reduced our ability to detect CMC/IMC abnormalities. However, clear abnormalities were still detected, even using the less affected hand.

## 5 Conclusion

In summary, young people with dystonia show abnormal patterns of CMC and IMC compared with typically developing children. The current findings demonstrate that some physiological features are common across different dystonia aetiologies (e.g. enhanced low-frequency IMC) while other features clearly differ (e.g. beta-CMC modulation following peripheral stimulation). This study provides evidence that sensorimotor processing and task-related cortical oscillatory activities are abnormal in Acquired as well as Idiopathic/Genetic dystonia, but that the nature of the abnormality is different. Further delineation and understanding of such similarities and differences is vital if we are to address the differential response to DBS seen between these groups (Neychev et al., 2011) and if we are to develop and/or modify therapies to optimize clinical benefit.
