# Supplementary material for: Abnormal patterns of corticomuscular and intermuscular coherence in childhood dystonia
Source: Clin Neurophysiol. 2020 Apr;131(4):967–77. doi: 10.1016/j.clinph.2020.01.012 (PMC7083222; doi:10.1016/j.clinph.2020.01.012)
Supplement: Supplementary data 1 [file mmc1.docx]

**Supplementary Material for:**

**Abnormal Patterns of Corticomuscular and Intermuscular coherence in childhood dystonia**

Verity M McClelland, Zoran Cvetkovic, Jean-Pierre Lin, Kerry R Mills, Peter Brown

**Supplementary Table S1** – details of cortical evoked potential (EP) for each subject.

| **Subject Number** | **Group** | **EP Peak latency (ms)** | **EP peak to peak amplitude (µV)** |
| --- | --- | --- | --- |
| 1 | Control | 32 | 9.1 |
| 2 | Control | 33 | 3.2 |
| 3 | Control | 34 | 6.8 |
| 4 | Control | 32 | 4.7 |
| 5 | Control | 30 | 7.0 |
| 6 | Control | 32 | 5.9 |
| 7 | Control | 41 | 3.9 |
| 8 | Control | 42 | 8.8 |
| 9 | Control | 33 | 3.5 |
| 10 | Control | 33 | 7.0 |
| 11 | Control | 28 | 11.4 |
| 12 | Control | 40 | 6.3 |
| 13 | Control | 78 | 9.9 |
|  | Mean +/- SD for Controls | 37.5 +/- 12.9 | 6.7 +/-2.6 |
| 14 | Idiopathic/Genetic | 39 | 11.7 |
| 15 | Idiopathic/Genetic | 37 | 12.8 |
| 16 | Idiopathic/Genetic | 37 | 1.5 |
| 17 | Idiopathic/Genetic | 35 | 6.3 |
| 18 | Idiopathic/Genetic | 40 | 9.1 |
|  | Mean for +/- SD Idiopathic/Genetic | 37.6 +/- 1.9 | 8.3 +/- 4.5 |
| 19 | Acquired | 37 | 5.1 |
| 20 | Acquired | 23 | 10.3 |
| 21 | Acquired | 36 | 6.4 |
| 22 | Acquired | 46 | 6.5 |
| 23 | Acquired | 38 | 11.6 |
| 24 | Acquired | 65 | 3.3 |
| 25 | Acquired | 36 | 3.6 |
| 26 | Acquired | 67 | 8.7 |
| 27 | Acquired | 44 | 3.6 |
| 28 | Acquired | 43 | 4.5 |
| 29 | Acquired | 39 | 8.7 |
|  | Mean +/- SD for Acquired | 43.1 +/-12.8 | 6.6 +/- 2.9 |

**Supplementary Table S2** – Individual coherence findings.

|  |  |  | **FDI EEGD**  **Corticomuscular Coherence** | | | **FExt EEGD**  **Corticomuscular Coherence** | | | **FDI FExt**  **Intermuscular Coherence** | | |
| --- | --- | --- | --- | --- | --- | --- | --- | --- | --- | --- | --- |
| **Case No.** | **Group** | **Age at study** | **Baseline** | **Post-stimulus** | **Peak Freq (Hz)** | **Baseline** | **Post-stimulus** | **Peak Freq (Hz)** | **Baseline** | **Post-stimulus** | **Peak Freq (Hz)** |
| 1 | Control | 16 | Y | Y | 14 | Y | Y | 30 |  | Y | 14 |
| 2 | Control | 18 |  | Y | 36 |  |  | 22 |  | Y | 24 |
| 3 | Control | 16 | Y | Y | 18 | Y | Y | 18 |  | Y | 18 |
| 4 | Control | 15 |  | Y | 24 | Y | Y | 26 |  | Y | 26 |
| 5 | Control | 16 |  | Y | 36 | Y | Y | 20 | Y | Y | 24 |
| 6 | Control | 18 |  | Y | 22 |  | Y | 24 |  | Y | 20 |
| 7 | Control | 15 | Y | Y | 26 | Y | Y | 26 | Y | Y | 24 |
| 8 | Control | 13 | Y | Y | 20 | Y | Y | 20 | Y | Y | 22 |
| 9 | Control | 17 | Y | Y | 20 | Y | Y | 22 | Y | Y | 18 |
| 10 | Control | 13 |  | Y | 36 |  | Y | 30 |  | Y | 20 |
| 11 | Control | 12 |  |  | 20 |  | Y | 30 |  | Y | 36 |
| 12 | Control | 16 | Y | Y | 20 | Y | Y | 20 | Y | Y | 20 |
| 13 | Control | 14 | Y | Y | 36 | Y | Y | 32 | Y | Y | 32 |
|  | **Summary for Control group** |  | **7/13**  **(54%)** | **12/13**  **(92%)** | **Median 22**  **Range 14-36**  **IQR 16** | **9/13**  **(69%)** | **12/13**  **(92%)** | **Median 24**  **Range 18-32**  **IQR 10** | **6/13**  **(46%)** | **13/13**  **(100%)** | **Median 22**  **Range 14-36**  **IQR 6** |
| 14 | Idiopathic/Genetic | 12 |  |  | 30 |  |  | 28 |  | Y | 18 |
| 15 | Idiopathic/Genetic | 13 |  | Y | 24 | Y | Y | 24 | Y | Y | 22 |
| 16 | Idiopathic/Genetic | 18 | Y | Y | 22 |  | Y | 30 | Y | Y | 28 |
| 17 | Idiopathic/Genetic | 12 |  |  | 30 |  |  | 26 | Y | Y | 18 |
| 18 | Idiopathic/Genetic | 17 |  | Y | 24 |  | Y | 24 | Y | Y | 24 |
|  | **Summary for Idiopathic/Genetic group** |  | **1/5**  **(20%)** | **3/5**  **(60%)** | **Median 24**  **Range 22-30**  **IQR 7** | **1/5**  **(20%)** | **3/5**  **(60%)** | **Median 26**  **Range 24-30**  **IQR 5** | **4/5**  **(80%)** | **5/5**  **(100%)** | **Median 22**  **Range 18-28**  **IQR 8** |
| 19 | Acquired | 12 |  | Y | 30 |  | Y | 34 | Y | Y | 26 |
| 20 | Acquired | 15 |  | Y | 18 | Y | Y | 26 |  | Y | 24 |
| 21 | Acquired | 15 |  | Y | 20 |  | Y | 20 | Y | Y | 20 |
| 22 | Acquired | 18 |  | Y | 16 | Y | Y | 22 | Y | Y | 22 |
| 23 | Acquired | 15 | Y | Y | 22 | Y | Y | 20 | Y | Y | 22 |
| 24 | Acquired | 17 |  |  | 30 |  | Y | 36 | Y | Y | 30 |
| 25 | Acquired | 17 | Y |  | 18 |  |  | 36 | Y | Y | 30 |
| 26 | Acquired | 14 |  | Y | 18 | Y | Y | 20 |  | Y | 26 |
| 27 | Acquired | 18 | Y | Y | 26 |  |  | 20 |  | Y | 26 |
| 28 | Acquired | 13 |  | Y | 24 | Y | Y | 20 | Y | Y | 20 |
| 29 | Acquired | 14 |  |  | 24 |  | Y | 36 | Y | Y | 26 |
|  | **Summary for Acquired Group** |  | **3/11**  **(27%)** | **8/11**  **(73%)** | **Median 22**  **Range 16-30**  **IQR 8** | **5/11**  **(45%)** | **9/11**  **(82%)** | **Median 22**  **Range 20-36**  **IQR 16** | **8/11**  **(73%)** | **11/11**  **(100%)** | **Median 26**  **Range 20-30**  **IQR 4** |

Y – indicates those participants showing significant coherence

FDI – First dorsal interosseous

FExt – Forearm extensors

EEGD – dominant hemisphere EEG

IQR – interquartile range

**Supplementary Table S3** – Acquired patients showing different patterns of beta-ERD (Event Related Desynchronisation) and CMC (corticomuscular coherence) modulation. Subject numbers are the same as those in the other tables.

| **Normal beta-ERD**  **Normal CMC modulation** | **Normal beta-ERD**  **Abnormal CMC modulation** | **Abnormal beta-ERD**  **Normal CMC modulation** | **Abnormal beta-ERD**  **Abnormal CMC modulation** |
| --- | --- | --- | --- |
| 19 | 26 | 22 | 20 |
| 21 | 27 | 24 |  |
| 23 |  |  |  |
| 25 |  |  |  |
| 28 |  |  |  |
| 29 |  |  |  |

**Supplementary Table S4a**

Median level of muscle contraction for each group expressed as % of MVC (maximum voluntary contraction).

|  | **Level of contraction as % MVC** | | |  |  |
| --- | --- | --- | --- | --- | --- |
|  | **Controls** | **Idiopathic/Genetic** | **Acquired** | **Kruskal Wallis H** | **P value** |
| **FDI** | 1.63 | 4.62 | 3.07 | 13.467 | 0.001 |
| **FExt** | 2.14 | 5.03 | 3.97 | 9.239 | 0.01 |

**Supplementary Table S4b**

Statistical comparisons between groups of the levels of EMG shown in Table 3a.

| **Muscle** | **Comparison** | **Mann-Whitney U** | **P value** |
| --- | --- | --- | --- |
| **FDI** | Controls vs Idiopathic/Genetic | 10.0 | 0.026 |
|  | Controls vs Acquired | 14.0 | 0.001 |
|  | Idiopathic/Genetic vs Acquired | 13.0 | 0.115 |
| **FExt** | Controls vs Idiopathic/Genetic | 21.0 | 0.002 |
|  | Controls vs Acquired | 14.0 | 0.075 |
|  | Idiopathic/Genetic vs Acquired | 26.0 | 0.913 |

FDI – First dorsal interosseous

FExt – Forearm extensors

**Supplementary Table S5a**

Spearman Correlation analysis to look for relationship between individual levels of beta-CMC and IMC and either a) level of EMG (expressed as percentage of MVC) or

b) contraction variability (expressed as Coefficient of Variation (CV) of rectified EMG).

| **EMG parameter** | **Coherence** | **Spearman Rho** | **p** |
| --- | --- | --- | --- |
| % MVC FDI | FDI:EEGD beta CMC | -0.070 | 0.717 |
| % MVC FExt | FExt: EEGD beta CMC | 0.073 | 0.705 |
| % MVC FDI | FDI:FExt beta IMC | 0.264 | 0.167 |
| % MVC FExt | FDI:FExt beta IMC | 0.195 | 0.310 |
| CV of Rectified FDI EMG | FDI:EEGD beta CMC | -0.005 | 0.979 |
| CV of Rectified FExt EMG | FExt: EEGD beta CMC | 0.087 | 0.655 |
| CV of Rectified FDI EMG | FDI:FExt beta IMC | 0.108 | 0.576 |
| CV of Rectified FExt EMG | FDI:FExt beta IMC | 0.101 | 0.602 |

FDI – First dorsal interosseous, FExt – Forearm extensors, EEGD – dominant hemisphere EEG,

MVC - Maximum voluntary contraction, CMC – corticomuscular coherence

IMC – intermuscular coherence, CV – coefficient of variation

**Supplementary Table S5b**

Spearman Correlation analysis to look for relationship between individual levels of low frequency IMC and either a) contraction variability (expressed as Coefficient of Variation (CV) of rectified EMG) or b) level of EMG (expressed as percentage of MVC). Note for (b) partial correlation was performed controlling for potential confounding effect of the difference in both MVC and IMC between groups.

| **EMG parameter** | **Coherence** | **Spearman Rho** | **p** |
| --- | --- | --- | --- |
| CV of Rectified FDI EMG | FDI:FExt low freq IMC | -0.171 | 0.375 |
| CV of Rectified FExt EMG | FDI:FExt low freq IMC | 0.119 | 0.540 |
| % MVC FDI | FDI:FExt low freq IMC | 0.122 | 0.535 |
| % MVC FExt | FDI:FExt low freq IMC | -0.079 | 0.689 |

FDI – First dorsal interosseous, FExt – Forearm extensors, EEGD – dominant hemisphere EEG,

MVC - Maximum voluntary contraction, CMC – corticomuscular coherence

IMC – intermuscular coherence, CV – coefficient of variation

**Sub-group Analyses within Acquired Dystonia Group**

Comparing the children with generalised dystonia-dyskinesia due to cerebral palsy (CP) (n=5) to those with a predominantly generalised dystonia phenotype (n=3), there was a trend towards higher levels of beta-CMC in the early post-stimulus period in the dystonia-dyskinesia CP group (Mann Whitney U=15.0, p=0.118) and the strength of modulation of beta-CMC from pre-stimulus to early post-stimulus was significantly higher in the dystonia-dyskinesia CP group (Mann Whitney U=8.0, p=0.016). (The children with asymmetric dystonia were not included in this analysis).

However, it is difficult to tease apart the role of the phenotype versus the aetiology. Comparing the children with dystonic-dyskinetic cerebral palsy (n=5) to those with a idiopathic/genetic dystonia (n=5, including 2 patients with predominantly generalised dystonia phenotype and 2 with additional myoclonic or dyskinetic movements) there was again a trend towards higher levels of beta CMC in the early post-stimulus period in the CP group (Mann-Whitney U = 28.0, p=0.105) and a significantly stronger modulation of CMC from baseline to early post-stimulus in the CP group (Mann-Whitney U = 19.0, p=0.019).

No trends were observed in the patterns of CMC in relation to location of MRI abnormalities (Basal ganglia or cortex).

Cautious interpretation is needed in view of the smaller numbers in these sub-group analyses, but the observations suggest that there may be differences in patterns of beta-CMC and its modulation across different aetiologies and/or phenotypes of dystonia. Further, larger studies in this field are therefore warranted.

**Coherence and Phase Spectra**

The phase of the coherence can be assessed where significant IMC is present across a range of frequencies, since a change in phase with frequency is required to demonstrate a non-zero phase difference. The slope of the phase-frequency plot for each subject was reviewed to confirm a linear relationship across the relevant frequencies. The slope of the phase-frequency plot where IMC was significant was tested to confirm that this was significantly different from zero (p<0.05) using linear regression.

**Supplementary Figure S1** – **Phase spectra.**

Example data from two individual patients (Left = subject 21, Right = subject 23) showing the intermuscular coherence spectra between FDI and FExt (A and B) and the respective phase spectra (C and D). (Rads = radians). A broadly linear phase-frequency relationship is seen across the frequency ranges in which significant intermuscular coherence is seen. The slope of the regression line across the relevant frequencies was significantly different from zero. Dashed orange line indicates 95% confidence level for significant coherence. Note difference scales in A and B due to stronger IMC in subject 23.

**Event Related Changes in EEG Power**

**Supplementary Figure S2 – Changes in EEG power over time.**

(A-C) % change in beta band power (14-36Hz) and (D-F) % change in alpha-theta band power (4-12Hz) with respect to baseline for EEG recorded over sensorimotor cortex contralateral to hand performing the task. Each coloured line shows data from a single subject. Mean power in respective band is plotted for 500ms overlapping windows in 50ms steps. Time-scale refers to mid-point of time window. Arrow shows time of stimulus. G-I: Beta/Alpha-theta ratio over time. Note the different time scale as the power changes are calculated with respect to baseline in A-F so baseline period is not shown. Two children in the Acquired dystonia group had very high Beta/Alpha-theta ratios and so are not plotted in I. J: Equivalent data to I but including all 11 children - note different magnitude scale compared with G-I.
